# Thunderclap Headache: A Primary Symptom of a Steroid-Responsive Encephalopathy with Autoimmune Thyroiditis

**DOI:** 10.1155/2021/5517934

**Published:** 2021-05-12

**Authors:** Naman Zala, Lena Wirth, Berit Jordan, Hagen Meredig, Timolaos Rizos

**Affiliations:** ^1^Department of Neurology, Heidelberg University Hospital, Im Neuenheimer Feld 400, Heidelberg 69120, Germany; ^2^Department of Neuroradiology, Heidelberg University Hospital, Im Neuenheimer Feld 400, Heidelberg 69120, Germany

## Abstract

Thunderclap headache is frequently associated with serious intracranial vascular disorders and a usual reason for emergency department admissions. Association of thunderclap headaches with autoimmune disorders, such as steroid-responsive encephalopathy with autoimmune thyroiditis (SREAT), is highly unusual. Here, we report a patient who presented with high-intensity headache of abrupt onset. Cerebrospinal fluid (CSF) analysis revealed moderate lymphocytic pleocytosis without evidence of infectious, neoplastic, or metabolic causes. Brain magnetic resonance imaging showed no specific pathologies, and examinations for neuronal antibodies in serum and CSF were negative. The medical history revealed that seven years before, an episode of an aseptic meningoencephalitis with remarkable response to steroids was present. Finally, increased levels of serum anti-TPO antibodies were identified, and against the background of a previous steroid-responsive aseptic meningoencephalitis, diagnosis of SREAT was highly probable. Methylprednisolone therapy was initiated, and the patient recovered completely. In particular, because most SREAT patients respond very well to steroids, this case underlines the importance of taking SREAT into consideration during the assessment of a high-intensity headache of abrupt onset.

## 1. Introduction

A high-intensity headache of abrupt onset (thunderclap headache) is a very common reason for seeking urgent medical advice [1]. By definition, thunderclap headache is of abrupt onset, reaching maximum intensity in less than 1 minute and lasting for at least 5 minutes or more [2]. Thunderclap headache is frequently associated with serious intracranial vascular disorders, including all types of intracranial hemorrhage (epidural, subdural, subarachnoid, or intracerebral hemorrhage), cerebral venous thrombosis, unruptured or thrombosed vascular malformation (mostly aneurysm), arterial dissection (intra- and extracranial), reversible cerebral vasoconstriction syndrome (RCVS), and pituitary apoplexy [3]. Other causes of thunderclap headache are meningitis, colloid cysts, spontaneous intracranial hypotension, and acute sinusitis (particularly with barotrauma) [2, 3]. A randomized population-based prospective study estimated an annual incidence of 43 cases per 100,000 adults [4].

In contrast to thunderclap headache, steroid-responsive encephalopathy with autoimmune thyroiditis (SREAT) is a rare disease, affecting only approximately 2.1 in 100.000 people [5]. SREAT is an autoimmune disorder presenting with neurological and neuropsychiatric symptoms and elevated titers of antithyroid antibodies. By definition, SREAT is associated with subacute onset of encephalopathy, presence of thyroid antibodies (antithyroid peroxidase antibodies and antithyroglobulin antibodies), and proven neurological improvement after immunotherapy, in the absence of other neural autoantibodies [6]. It is recognized as a rare disease with diagnosis based on a rigorous clinical assessment and comprehensive testing for well-characterized neuronal antibodies to exclude other potential causes of encephalopathy [7]. The clinical presentation of patients with SREAT is highly variable, encompassing a broad spectrum of complaints and symptoms [8]. Particularly due to the broad variety of clinical manifestations and subtlety of symptoms, which may be acute or chronic [9], diagnosing SREAT remains a major challenge.

Although headache and, in particular, migraine-like headaches are reported in SREAT patients, sudden and severe headache (i.e., thunderclap headache) as the first symptom has not yet been documented. Thus, we here report a case with thunderclap headache being the initial symptom of a relapsing SREAT.

## 2. Case Report

A 79-year-old Caucasian female with known history of hypertension, diabetes mellitus type 2, hypothyroidism, and mild renal impairment was admitted to our emergency department (ED) because of a sudden onset of severe bifrontal headache. The headache was rated by the patient as 8/10 on the numeric rating scale for pain. Along with that, she complained of difficulties in concentration. There were no preceding trauma and no history of migraine or other intermittent headaches. The initial neurological examination was normal. Cranial computer tomography showed minor microangiopathic and major macroangiopathic changes and strio-pallido-dentate calcifications but no other pathologies. Standard laboratory results revealed a mild microcytic hypochrome anaemia (Hb: 9,9 g/dl, MCV: 77fl, and MCH: 25pg) and a mild impaired renal function (glomerular filtration rate: 48 ml/min). Metamizole (2 g/d) was administered, which led to significant improvement and discharge.

**Figure 1:**
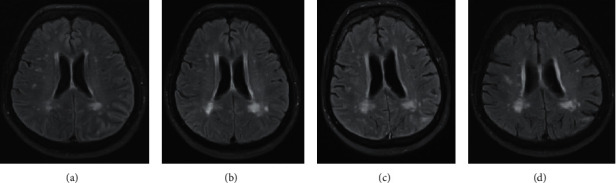
FLAIR images (a–d) showing progressive diffuse signal abnormality involving the white matter of both cerebral hemispheres over 7-year period (a–c) with gradual resolving after steroid treatment (d). (a) June 2012. (b) October 2012. (c) July 2019. (d) February 2020.

Within 24 hours of being discharged, the patient experienced difficulty in speaking, and she was referred again to our ED. The neurological examination at admission still remained normal, especially with no signs of aphasia or dysarthria. Within hours, the patient became delirious with partially aggressive behavior accompanied by optic hallucinations. Brain magnetic resonance imaging (MRI) showed minor microangiopathic changes along with small left temporoparietal postischemic defects. Moreover, bilateral strio-pallido-dentate calcifications and minor artifacts in the left occipital region related to calcification of the tentorium were noticed. A cerebrospinal fluid (CSF) analysis showed moderate lymphocytic pleocytosis (48 cells/*μ*l; total protein: 0.695 g/l; glucose: 78.0 mg/dl; lactate: 1.8 mmol/l). Under the suspicion of an infectious meningoencephalitis, broad-spectrum antimicrobial and antiviral therapy with ceftriaxone and acyclovir was initiated. The microbiological analysis of CSF showed no evidence of any bacterial or fungal pathogen. The polymerase chain reaction tests for herpes simplex 1&2 DNA, varicella zoster DNA, cytomegalovirus DNA, Ebstein–Barr virus DNA, enterovirus RNA, as well as specific anti-Borrelia burgdorferi antibodies (IgG & IgM) were negative. We then considered an ictal phenomenon as a possible differential diagnosis for the presenting symptoms and started antiepileptic therapy with valproic acid (900 mg/d). Nevertheless, the electroencephalography (EEG) showed no signs of epileptiform discharges or any abnormal background EEG frequencies. Even after an intravenous antimicrobial therapy for seven days, the patient's clinical state remained to a large extent unchanged. The neuropsychological screening (Montreal Cognitive Assessment: 13/30 points) showed a considerable cognitive deterioration. We decided to perform a follow-up CSF analysis to rule out the presence of paraneoplastic neuronal antibodies as a probable cause of the meningoencephalitis. Again, a moderate lymphocytic pleocytosis was observed (34 cells/*μ*l; total protein: 0.609 g/l; glucose: 59.0 mg/dl; lactate: 1.7 mmol/l), and a serological and CSF analysis provided no signs of any pathogens. Antineuronal antibody analysis revealed normal results for anti-Hu, Ri, ANNA-3, Yo, Tr/DNER, Ma/Ta, GAD65, amphiphysin, aquaporin-4, MOG, glutamate receptors (Type NMDA and AMPA), GABA A/B receptors, LGI1, CASPR2, IgLON5, ZIC4, DPPX, antimyelin, glycin receptors, mGluR1, mGluR5, Rho-GTPase activating protein 26, ITPR1, Homer 3, recoverin, neurochondrin, GluRD2, and flotillin ½. In conjunction to this, the CSF biomarkers for dementia showed elevated levels of tau protein (478 pg/ml, norm: <450 pg/ml) and phospho-tau protein (90 pg/ml, norm: <61 pg/ml) as well as a reduced Aß ratio (0.38, norm: >0.5). On the 10th day after hospitalization, the symptoms remained mostly unchanged, and follow-up brain MRI was conducted. Compared to the first MRI, progressive leptomeningeal and sulcal T2/fluid-attenuated inversion recovery (FLAIR) hyperintensities parieto-occipital on both sides and temporoparietal on the left side were seen. Moreover, increasing unspecific subcortical and periventricular T2-FLAIR hyperintensities were observed (Figure 1). Careful reevaluation of the medical history revealed a preceding episode of aseptic meningoencephalitis seven years before with comparable clinical findings. Although an extensive diagnostic analysis at that occasion showed no conclusive cause of the meningoencephalitis, there were remarkable effects of the steroid therapy. Based on this, we recommended a probatoric treatment with steroids. However, because the medication with steroids initially caused severe worsening of neuropsychiatric symptoms seven years ago, the patient's legal representatives rejected this recommendation and urged to discharge the patient which was then done.

Five days later, our patient was again referred to our ED because of the worsening of the neuropsychiatric symptoms, in particular, the visual and the auditory hallucinations. We again conducted a CSF analysis which showed no relevant changes (lymphocytic pleocytosis: 43 cells/*μ*l; protein: 0.457 g/l; glucose: 59 mg/dl; and lactate 1.8 mmol/l; and no evidence of intrathecal IgG or IgM synthesis). Although the patient had a known history of hypothyroidism, autoimmunological laboratory diagnostic regarding thyroid antibodies was never conducted. Taking the aforementioned medical history into consideration, we augmented previous laboratory tests with autoimmune thyroid antibodies which was not done seven years ago. Here, antithyroid peroxidase antibodies (anti-TPO antibodies) were markedly increased (344 IU/l, norm: <60IU/l). Nevertheless, the thyroid-stimulating hormone (TSH), thyroxine (T4), and the triiodothyronine (T3) were within normal ranges. The ultrasound of the thyroid gland revealed no clear evidence of thyroiditis, and the patient was clinically in an euthyroid state.

Together with the history of steroid-responsive aseptic meningoencephalitis in 2012, diagnosis of SREAT was highly probable, and a high-dose immunosuppressive therapy with intravenous methylprednisolone (1000 mg i.v. for 3 days, with tapering oral dose at a rate of 10 mg/week thereafter and eventual maintenance dose of 10 mg/day) was commenced. Under this treatment regime, neuropsychiatric symptoms showed clear and fast improvements. Visual and auditory hallucinations resolved completely after 3 days. Also, the neuropsychological screening (Montreal Cognitive Assessment: 19/30 points) showed improvement. Three months later, the patient was seen in our outpatient department. The CSF analysis at that time was completely normal (3 cells/*μ*l; protein: 0.52 g/l; glucose: 72.0 mg/dl; lactate: 1.88 mmol/l). In addition to this, the neuropsychological deficits had improved remarkably. A follow-up brain MRI showed clear signs of improvement. Maintenance dose of oral steroids was further reduced to 5 mg/day and continued to prevent a further relapse of SREAT.

## 3. Discussion and Conclusion

Steroid-responsive encephalopathy with autoimmune thyroiditis is a rare but very serious illness. Importantly, no SREAT-specific clinical, laboratory, or radiological findings are existent, and the disease is often missed, underdiagnosed, or diagnosed with a substantial time delay. Hence, it can be assumed that, because of low overall awareness and due to limited diagnostic workup in patients with otherwise unexplained encephalopathy, the incidence of SREAT is underestimated.

Clinically, both sudden onset of focal neurological symptoms and gradually increasing neurological complaints are described. A Chinese study identified cognitive impairment and psychiatric symptoms as the most common presentation [10]. However, in a review by Ferracci and colleagues, seizures and myoclonus were among the most frequently reported events [5]. Two types of SREAT have been suggested. The first being relapsing/remitting, also referred to as vasculitic type, which manifests with encephalopathy and stroke-like episodes [9]. The other is a diffuse progressive type, which has an insidious onset and a progressive course with occasional fluctuations, and manifests with psychiatric symptoms (including confusion and hallucinations), dementia, and impairment of the sleep-wake cycle [9]. Either type may also present with tremor, myoclonus, seizures, stupor, or coma [9]. Importantly, the presence of thyroid autoantibodies and steroid responsiveness of symptoms is deemed mandatory for the final diagnosis (Table 1). Nevertheless, titers of anti-TPO antibodies do not clearly correlate with the severity of the disease or improvement of clinical symptoms [11].

Thunderclap headache in a patient without a history of migraine or other intermittent headaches being the primary symptom of a steroid-responsive encephalopathy with autoimmune thyroiditis represents a peculiarity of our case. In SREAT, headache may occur in up to 80% of the patients, but it is normally mild or periodic, not a presenting sign, and is usually attributed to previous migraine and hypothyroidism. The pathogenesis of SREAT is still unclear. Whether the existence of antithyroid antibodies only represents an epiphenomenon in the setting of SREAT or whether they represent a pathogenic factor is still unknown. It could be speculated that an associated inflammatory response may result in perivascular nociceptor activation which might have led to a thunderclap headache in our patient. Because of the broad number of possible differential diagnosis, including subarachnoid hemorrhages, associated with sudden severe headache, a thorough medical clarification is of utmost importance.

**Figure 2:**
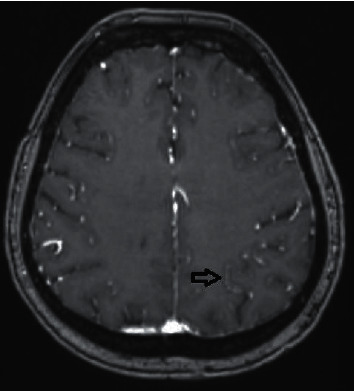
MRI from June 2012 showing hyperperfusion of the leptomeningeal arteries (black arrow).

There are reports of different MRI findings ranging from mild generalized cerebral atrophy, bilateral subcortical lacunar infarcts, generalized leukoencephalopathy, diffuse signal abnormality involving the white matter of both cerebral hemispheres, as well as cerebellar and cerebral peduncles in patients with SREAT [12]. Our patient had documented evolving MRI changes spanning 7 years of diffuse leukoencephalopathy, mild cortical atrophy, and increased T2/FLAIR white matter and sulcal hyperintensities as well as leptomeningeal enhancements (Figure 2) illustrating a wide range of MRI changes that can be seen in a single case of SREAT.

The optimal dose of oral steroids is not known and is primarily based on the individual's response to treatment. Although steroid responsiveness represents a SREAT defining factor, more aggressive and long-term immunosuppressive therapy is needed in some cases [13]. In our patient, steroid therapy led to a complete remission of symptoms, and CSF results were normalized.

To conclude, the present case underlines that awareness for SREAT is important when evaluating patients with acute onset of high-intensity headache associated with neuropsychiatric symptoms, particularly because most patients respond very well to steroids.

## Figures and Tables

**Table 1:** Diagnostic criteria for SREAT [6, 14].

1.	Encephalopathy characterized by cognitive impairment, neuropsychiatric symptoms, myoclonus, partial or generalized tonic-clonic epileptic seizures, and focal neurological deficits
2.	Euthyroid, subclinical, or mild-to-moderate clinical hypothyroidism (with corresponding TSH levels)
3.	Normal brain MRI or with nonspecific abnormalities
4.	Presence of antithyroperoxidase antibody and possibly antithyroglobulin antibody and/or anti-thyroid-stimulating hormone receptor stimulating antibody
5.	Absence of well-characterized neuronal antibodies in serum and CSF
6.	No evidence of infectious, toxic, neoplastic, or (other than thyroid-associated) metabolic disease
7.	Complete or near complete remission with steroid therapy

## Data Availability

No data were used to support this case report.
